# *Anaplasma phagocytophilum* Infection in *Ixodes ricinus*, Bavaria, Germany

**DOI:** 10.3201/eid1406.071095

**Published:** 2008-06

**Authors:** Cornelia Silaghi, Jérémie Gilles, Michael Höhle, Volker Fingerle, Frank Thomas Just, Kurt Pfister

**Affiliations:** * Ludwig-Maximilians-University, Munich, Germany; 1 Current affiliation: University of Kentucky, Lexington, Kentucky, USA.

**Keywords:** Anaplasma phagocytophilum, prevalence, ticks, Ixodes ricinus, molecular epidemiology, Bavaria, Germany, dispatch

## Abstract

*Anaplasma phagocytophilum* DNA was detected by real-time PCR, which targeted the *msp2* gene, in 2.9% of questing *Ixodes ricinus* ticks (adults and nymphs; n = 2,862), collected systematically from selected locations in Bavaria, Germany, in 2006. Prevalence was significantly higher in urban public parks in Munich than in natural forests.

*Anaplasma phagocytophilum*, an obligate intracellular bacterium, causes a febrile disease in ruminants and granulocytic anaplasmosis in dogs, horses, and humans (*1*). A reorganization of the order Anaplasmataceae reclassified *Ehrlichia equi*, *E. phagocytophila,* and the human granulocytic ehrlichiosis (HGE) agent to the single species *A. phagocytophilum* (*2*), which in Europe is transmitted by the sheep tick, *Ixodes ricinus* (*3*). The agent is found among the *I. ricinus* population in Germany; average prevalence rates are 1% to 4.5% (*4*,*5*). The English Garden, a large (3.7-km^2^) public park in Munich (state of Bavaria, Germany), has been suggested in 2 previous studies as a focal point for *A. phagocytophilum* (*5*,*6*). We investigated *A. phagocytophilum* in questing ticks in urban areas of Munich and focused on seasonal and geographic effects on the prevalence.

## The Study

**Figure 1:**
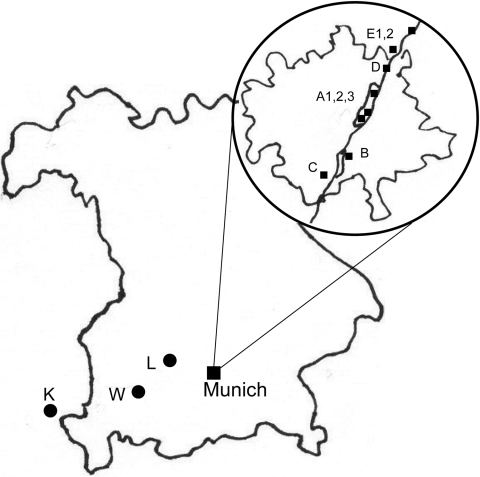
Location of collection sites. Large map, Bavaria, Germany; circled inset, city of Munich (with the Isar River). Sites in Munich area: A1, 2, 3, English Garden park; B, city park; C, D, E1, 2, riparian and deciduous forest; K, L, W, mixed forest areas outside of Munich.

The sampling consisted of 2 phases. First, to gain an overview on the occurrence of *I. ricinus,* we collected questing ticks by the flagging method at 8 locations (labeled A1, A2, A3, B, C, D, E1, E2) close to the Isar River in the Munich area from May through September 2006 (Figure 1). Sites A1 and A2 were located in the city center part of the English Garden, which is enclosed by roads and houses. The vegetation of this heavily frequented area consists of groomed lawns, bushes, and deciduous trees. Site A3 was located in the northern part of the Garden, where vegetation was maintained by gardening, but bushes and trees were denser and grassland less frequently cut. The site was also used for horseback riding. Site B was a landscaped public green in the southern part of the city with groomed lawns and deciduous trees. Sites C, D, E1, and E2 were periurban riparian and deciduous forests. Three natural mixed forest sites (K, L, W) outside of Munich were sampled once (Figure 1). Ticks were registered and frozen individually at –26°C; adults were identified to species level by standard taxonomic keys (*7*). In the second phase, DNA was extracted from randomly chosen ticks (as available, 30 females, males, and nymphs, respectively, per month per site) with the High Pure PCR Template Preparation Kit (Roche, Mannheim, Germany) according to manufacturer’s instructions with modifications. In individual 1.5-mL tubes, each tick was crushed mechanically with a metal spatula; sterile water (200 μL) was added, and the sample was kept overnight in a 55°C water bath for complete tissue lysis. At the beginning and end of each extraction line, a negative control was added. Quality and quantity of extracted DNA were tested with a spectrophotometer (NanoDrop ND-1000, PeqLab, Erlangen, Germany). A real-time PCR targeting the *msp2* gene of *A. phagocytophilum* (*8*) was performed with modifications in a Bio-Rad iCycler iQ (Bio-Rad, Munich, Germany). In a reaction volume of 25 μL, the HotStarTaq Buffer Set was used with 1.25 U HotStarTaq Polymerase (both QIAGEN, Hilden, Germany), 6 mmol/L MgCl_2_, 200 μmol/L each dNTP, 900 nmol/L each primer (ApMSP2f /ApMSP2r [*8*]), 125 nmol/L probe ApMSP2p-HEX (*8*), and 5.0 μL template DNA. Cycling conditions were as follows: initial activation (95°C, 15 min), 50 cycles denaturation (94°C, 15 s), and annealing–extension (60°C, 60 s). The original protocol was also used for part of the samples (*8*). Thirty-one DNA extracts, positive in real-time PCR, were amplified in a Thermocycler GeneAmp PCR System 2700 (Applied Biosystems, Weiterstadt, Germany) with a nested PCR (*9*) targeting the 16S rRNA gene, amplification of which is necessary to differentiate closely related strains (*8*). Negative and known positive controls were always included. After the final products were analyzed by 1.5% agarose gel electrophoresis and purified with the QIAquick PCR Purification Kit (QIAGEN) according to manufacturer’s instruction, the 497-bp fragments, without flanking primers, were sent for sequencing to MWG, Martinsried, Germany. The results were evaluated with ChromasLite (www.technelysium.com.au/chromas_lite.html), sequence homology searches made by BLASTn analysis of GenBank sequences (www.ncbi.nlm.nih.gov.library.vu.edu.au/BLAST), and multiple alignments (www.ebi.ac.uk/clustalw/index.html). The effects of month, location, stage, and sex of ticks on probability of infection were investigated with logistic regressions by using R version 2.5.0 (*10*); p<0.05 was regarded as significant. Due to low prevalence of *A. phagocytophilum*, odds ratios were interpretable as relative risks (RR). We calculated monthly prevalence with a weighted analysis, taking into account the sampling design: phase 1, a random sample, is stratified by sex, and in phase 2, a fixed number was drawn monthly at random within each sex stratum. Estimates were based on the Horvitz-Thompson estimator and corresponding 95% confidence intervals (CIs) computed by parametric bootstrap conditioning on phase 1 sample sizes (*11*).

A total of 9,507 ticks (4,932 adults, 3,573 nymphs, and 1,001 larvae) were collected, and adults were identified as *I. ricinus*. Real-time PCR was performed for 2,862 ticks (Table; online Appendix Table, available from www.cdc.gov/EID/content/14/6/972-appT.htm). With the modified protocol, atypical amplification occurred in ≈10% of samples, whereas with the original protocol, which had been tested on *I. scapularis* ticks, no amplification occurred. This difference suggests unspecific reactions in the modified protocol. *A. phagocytophilum* was detected in 5.67% of females, in 4.00% of males, and in 1.14% of nymphs (Table). The overall prevalence was 2.9% (95% CI 2.3%–3.5%). Significantly more females and males were infected than nymphs (RR = 4.906 for females, RR = 3.439 for males; p<0.001). Prevalence was significantly higher in the city parks (A1, A2, A3, B) than in natural forest areas (C, D, E1, E2, K, L, W; RR = 0.368, p<0.001). Prevalence was significantly lower in the riparian forest, Isarauen (E1, E2) in the north of Munich, than in the English Garden (A1, A2, A3) (RR = 0.314, p<0.001). Variations among the collection months, ranging from 0% to 20% for females and males and from 0 to 9.1% for nymphs (online Appendix Table, available from www.cdc.gov/EID/content/14/6/972-appT.htm), were not significant (p = 0.40).

**Table:** Total *Anaplasma phagocytophilum*–infected *Ixodes ricinus* ticks per site, southern Germany, 2006*

Study site	No. infected ticks/no. total ticks (%)		
	Females	Males	Nymphs
A1	11/87 (12.64)	5/88 (5.68)	3/104 (2.88)
A2	10/149 (6.71)	12/153 (7.84)	3/150 (2.00)
A3	7/114 (6.14)	4/105 (3.81)	1/83 (1.20)
B	8/80 (10.00)	5/65 (7.69)	0/42
C	1/68 (1.47)	1/60 (1.67)	0/96
D	5/150 (3.33)	5/152 (3.29)	2/142 (1.41)
E1	3/92 (3.26)	1/101 (0.99)	2/114 (1.75)
E2	5/122 (4.10)	1/134 (0.75)	0/140
K	1/30 (3.33)	1/31 (3.23)	0/30
L	1/30 (3.33)	2/30 (6.67)	0/30
W	2/30 (6.67)	1/30 (3.33)	0/30
Total	54/952 (5.67)	38/949 (4.00)	11/961 (1.14)

*A1, A2, A3, English Garden in Munich; B, other park in Munich; C, D, E1, E2, periurban forest areas of Munich; W, K, L, forests outside of Munich (compare Figure 1).

**Figure 2:**
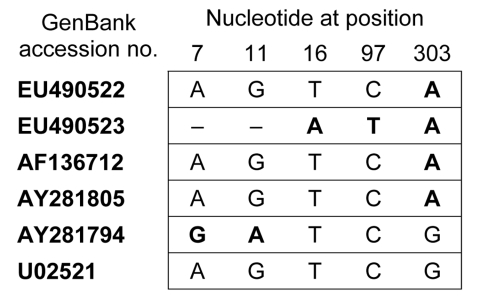
Comparison of the 497-bp sequences of *Anaplasma phagocytophilum* obtained from *Ixodes ricinus* ticks, Bavaria, Germany, 2006, in relation to selected GenBank sequences.

Alignment of the partial 16S rRNA gene sequences showed that 30 sequences were 100% identical (GenBank accession no. EU490522); 1 sequence differed in 2 nucleotide positions (accession no. EU490523). The 30 homologous sequences were 100% identical to *Ehrlichia* sp. Frankonia 2 when compared with GenBank sequences (Figure 2) of *Ehrlichia* sp. Frankonia 2, *A. phagocytophilum* isolate X7, *A. phagocytophilum* isolate P80, and the prototype sequence of the HGE agent (GenBank accession nos. AF136712, AY281805, AY281794, and U02521, respectively). For Frankonia 2 and *A. phagocytophilum* isolate X7, the remaining sequence differed in 1 nt position. All differed in 1 nt position from the prototype HGE agent and *A. phagocytophilum* isolate P80 and in 2 more nt positions from P80.

## Conclusions

Our results indicate that city parks of Munich may be focal points for *A. phagocytophilum*. Focal distribution depends mainly on mammalian reservoir hosts because of lack of transovarial transmission in ticks (*12*). Wood mice, yellow-necked mice, voles, roe, and red deer have been suggested as reservoirs in Europe (*13*,*14*). In the parks, a different reservoir host might be present. Large numbers of people and their domestic dogs pass through the parks, and the possibility of dogs acting as reservoirs for *A. phagocytophilum* should be investigated in further studies.

*Ehrlichia* sp. Frankonia 2 was first detected in adult ticks collected from domestic dogs in central Germany (*15*) and was later found in questing adults in Munich (*5*). However, neither *Ehrlichia* sp. Frankonia 2 nor the closely related *A. phagocytophilum* isolate X7 has been detected in humans or animals; thus, they can be regarded as strains of unknown pathogenicity. Future studies should aim at characterization of this strain and its possible role as a human or veterinary pathogen, as well as the identification of potential reservoir hosts in the city parks.
